# Upfront Treatment of FLT3-Mutated AML: A Look Back at the RATIFY Trial and Beyond

**DOI:** 10.3389/fonc.2020.562219

**Published:** 2020-12-22

**Authors:** Gina Keiffer, Kimberly L. Aderhold, Neil D. Palmisiano

**Affiliations:** Sidney Kimmel Cancer Center, Thomas Jefferson University, Philadelphia, PA, United States

**Keywords:** acute myeloid leukemia, FLT3, midostaurin, gilteritinib, first line

## Introduction

In April 2017, following the results of the RATIFY trial (1), midostaurin, a multikinase FLT3 inhibitor, became the first FDA approved targeted agent for the treatment of acute myeloid leukemia (AML) (2). The addition of midostaurin to standard induction therapy with anthracycline and cytarabine (7 + 3) rapidly became the new standard of care for treatment-naïve, fit patients with FLT3-mutated (FLT^mut+^) AML (3). More recently, gilteritinib, a selective FLT3 inhibitor, showed superiority to chemotherapy in the treatment of relapsed or refractory FLT^mut+^ AML (4).

With two FLT3 inhibitors now approved by the FDA—that is, the more selective gilteritinib and the less selective midostaurin—the question of which FLT3 inhibitor to use in combination with chemotherapy in the upfront setting has become the subject of much debate (5–7). Leukemia physicians are faced with the choice of using a more selective agent in the front line vs. reserving that agent for the time of relapse. Here, we evaluate the rationale for both approaches.

## Background

### What Is FLT3?

*Fms-like Tyrosine Kinase 3* (FLT3) is a type III receptor tyrosine kinase (RTK) expressed on hematopoietic stem cells that is well known to play a key role in hematopoietic expansion. Wild type FLT3 exists in the inactive monomeric conformation, which is maintained by the auto-inhibitory interaction of the juxtamembrane and internal kinase domains and inhibits ATP-binding. When FLT3 ligand binds the external domain, the receptor dimerizes, releasing of the inhibitory interaction and activating the kinase domain to undergo autophosphorolyation and, *via* signaling of the PI3K/AKT, MAPK and JAK/STAT pathways, promote cell survival and proliferation (8).

FLT3 is mutated in approximately 25–35% of patients with AML (9). Mutations occur either as internal tandem duplications (ITD), which occur in approximately 25% of AML, or as tyrosine kinase domain (TKD) mutations, which occur in approximately 7% of AML. ITD mutations are in-frame insertions which occur primarily in the juxtamembrane domain, disrupting the auto-inhibitory function of this domain and yielding constitutive FLT3 activation (8). It has long been understood that patients with FLT3-ITD mutation have significantly poorer survival than their FLT3-wild type counterparts (9–11) regardless of co-occurring mutations and other cytogenetic abnormalities (12). The pathologic significance of TKD mutations, which and are primarily point mutations, is less well understood with the exception of D835 mutations, an activating mutation which is also known to confer resistance to some FLT3 inhibitors.

The European Leukemia Net (ELN) classifies the prognostic import of FLT3-ITD mutation status based on the presence of co-occurring mutation in nucleophosmin 1 (NPM1) and the FLT3-ITD allelic ratio (AR). The FLT3-ITD AR is determined using DNA fragment analysis as the ratio of the area under the curve “FLT3-ITD” divided by the area under the curve “FLT3-wild type”. A low AR (FLT3-ITD^low^) is defined as <0.5 and a high AR (FLT3-ITD^high^) is defined as ≥0.5. Patients with FLT3-ITD mutation are characterized as favorable risk if they are FLT3-ITD^low^ and have a co-occurring NPM1 mutation. Patients with FLT3-ITD mutation are characterized as intermediate risk if they are FLT3-ITD^low^ and are NPM1 wild type (without other high-risk genetic lesions) or if they are FLT3-ITD^high^ and have a co-occurring NPM1 mutation. Patients with FLT3-ITD mutation are characterized as adverse risk if they are FLT3-ITD^high^ and are NPM1 wild type (13).

### What Are the Characteristics of FLT3 Inhibitors?

FLT3 inhibitors are classified both by selectivity of RTK binding (multikinase inhibitors vs. selective FLT3 inhibitors) and by site of binding FLT3 (type I vs. type II inhibitors). First generation FLT3 inhibitors, such as sorafenib, sunitinib and midostaurin, are unselective and bind multiple type III RTKs [e.g., platelet derived growth factors alpha and beta, stem cell factor receptor (KIT) and colony stimulating factor 1 receptor] and are therefore referred to as multikinase inhibitors. Second generation inhibitors, such as quizartinib, crenolanib and gilteritinib, are more selective and bind FLT3 only or a small number of additional RTKs (14).

Type I inhibitors bind FLT3 at the ATP-binding site in either the active or inactive conformation and therefore have activity in both ITD- and TKD-mutated patients. Type II inhibitors, conversely, bind the hydrophobic region adjacent to the ATP-binding site which is only accessible in the inactive conformation, thereby preventing FLT3 activation. TKD mutations, most notably D835 mutations, cause the FLT3 receptor to favor the active conformation thereby precluding binding of type II inhibitors (14). **Table 1** summarizes the classification of various FLT3 inhibitors.

**Table 1:** Summary of classification of FLT3 Inhibitors.

	Multikinase Inhibitors	Selective FLT3 Inhibitors
**Type I Inhibitors**Activity in both ITD- and TKD-mutations	MidostaurinLestauritinib**Sunitinib**	CrenolanibGilteritinib
**Type II Inhibitors**Activity in ITD-mutations only	SorafenibPonatinib	Quizartinib

**No longer under clinical investigation for treatment of FLT^mut+^ AML.

## Discussion: Which FLT3 Inhibitor Should be Used for Upfront Combination Therapy in FLT3-Mutated AML

Two FLT3 inhibitors have been approved for the treatment of FLT3-mutated AML: midostaurin and gilteritinib. Based on the results of the RATIFY trial, midostaurin was approved in combination with cytotoxic chemotherapy for the upfront treatment of FLT^mut+^ AML (2). In May, 2019, following early results of the ADMIRAL trial (4), the FDA approved gilteritinib for the treatment of relapsed or refractory FLT^mut+^ AML with a detectable FLT3 mutation (15). Since that time, promising early data has emerged regarding the use of gilteritinib in combination with 7 + 3 in the upfront setting (16).

The promising nature of these results prompted us to raise the question of whether upfront combination treatment with a multikinase inhibitor, such as midostaurin, or a selective FLT3 inhibitor, such as gilteritinib, is a more logical approach. While it may be argued that this debate is premature, particularly since a clinical trial is currently ongoing to address this exact question (NCT03836209) (17), we argue that it will not be long until the final results of gilteritinib combined with chemotherapy in treatment-naïve patients will be available; and we will then, without a randomized comparison, be forced to choose between these two approaches. Further, if these results remain positive, it may be hard to recruit to a trial randomizing these two agents due to lack of equipoise.

### The Case for Use of Midostaurin in Initial Induction Therapy in FLT3-Mutated AML

#### Data for Upfront Midostaurin: The RATIFY Trial

The RATIFY trial (1) was a multinational, double-blinded, placebo-controlled randomized trial evaluating the addition of midostaurin on days 8–21 to conventional chemotherapy (7 + 3) in treatment-naive FLT^mut+^ AML patients age 18–59 years old. Patients with both FLT3-ITD and FLT3-TKD mutations were included so long as mutant to wild type allelic ratio (the proportion of cells with mutant FLT3 to wild type FLT3) was ≥0.05. Patients were stratified by type of FLT3 mutation (TKD, ITD^high^ (AR >0.7) and ITD^low^ (AR 0.05–0.7). Note that the definition FLT3-ITD^low^ vs FLT3-ITD^high^ here differs from that used in the ELN risk stratification classification. If patients remained in remission following consolidation therapy, they could go on to receive midostaurin maintenance for up to 1 year or until allogeneic stem cell transplant (allo-SCT). The primary endpoint was overall survival (OS).

Seven hundred and seventeen patients with a median age of 47.9 years were randomized to midostaurin + chemotherapy (n=360) vs. placebo + chemotherapy (n=357). The types of FLT3 mutations were equally distributed between the placebo and midostaurin groups (overall, 47.6% ITD^low^, 29.8% ITD^high^, 22.6% TKD). Despite very similar complete response (CR) rates between the midostaurin (58.9%) and placebo (53.5%) groups, the median OS in the midostaurin group was 74.7 months vs. 25.6 months with placebo, and 4-year OS was 51.4 vs. 44.2%, respectively. The authors noted that the large difference in median OS was likely due to the tail of the survival curves for midostaurin + chemotherapy vs. placebo + chemotherapy falling just above and just below the 50% mark, respectively, and that a hazard ratio (HR) of 0.78 (95% CI 0.66–0.93) was more reflective of the clinical benefit of the addition of midostaurin to standard combination chemotherapy. Secondary endpoints of event free survival (EFS) and disease free survival (DFS) also favored the midostaurin group (8.2 months vs. 3.0 months and 26.7 months vs. 15.5 months, respectively).

The type of FLT3 mutation did not clearly impact overall survival [HR 0.65 FLT3-TKD (CI 0.39 – 1.08) vs. 0.81 FLT3-ITD^low^ (CI 0.6–1.11) vs. 0.80 FLT3-ITD^high^ (CI 0.57–1.12)] but when analyzed for EFS, the benefit of midostaurin was primarily seen in the TKD group.

Adverse effects were as expected for patients receiving induction and consolidation chemotherapy. Rates of anemia and rash were higher in the midostaurin group. More patients in the placebo group experienced nausea.

These data clearly support the addition of midostaurin to standard induction and consolidation therapy in FLT^mut+^ AML. No other FLT3 inhibitor has shown this degree of benefit in a phase III randomized clinical trial. The benefit of midostaurin on overall survival was seen across mutation type. Thus, the answer of which FLT3 inhibitor to use in the upfront setting is simple: midostaurin.

#### Upfront Multikinase Inhibitors May Inhibit Multiple Pathways in a Polyclonal Disease

Beyond the support for upfront use of midostaurin observed in the RATIFY trial, it has been suggested (6, 18) that the optimal sequencing of FLT3-inhibition may be to utilize multikinase inhibitor in the upfront setting when the disease is known to be polyclonal (19) and to save a more targeted FLT3 inhibitor for the time of relapse when the disease may have fewer clones and may be more dependent on FLT3 signaling. It is important to note that this suggestion is merely theoretical. At present, there are no available diagnostics to determine the number or relative impact of various malignant clones either at diagnosis or at relapse in AML. This theory is, however, does have some support.

As the authors of RATIFY note, the observed benefit of midostaurin in FLT3-ITD^low^ patients, where mutations other than FLT3 may function as drivers, may be due to the multitarget effects of midostaurin *via* inhibition of alternate or additional pathways, such as KIT. Additionally, it has been observed that midostaurin, although not effective as a single agent, does cause reduction of bone marrow blasts in both FLT3^mut+^ and FLT3 wild type AML (20). Conversely, samples from patients with relapsed AML with high FLT3^mut+^ allelic burden were more sensitive to FLT3 inhibition compared to samples obtained at diagnosis with lower FLT3^mut+^ AR (21). Thus, a strategy which uses a multikinase inhibitor upfront, while delaying the use of the more potent, selective inhibitor until relapse, may theoretically be beneficial for disease control, but this remains to be proven.

### The Case for Use of Gilteritinib Combined With Chemotherapy as Initial Induction Therapy for FLT3-Mutated AML

#### Problems With RATIFY

The rates of CR in the midostaurin and placebo groups were equivalent. Despite this, patients who received midostaurin experienced improved EFS, DFS and OS. Although, the survival curves overlap completely until approximately 9 months. This suggests that the benefit of midostuarin is not that it caused more remissions than placebo, but it caused deeper remissions. Furthermore, while FLT3 mutation type did not seem to impact response to midostaurin in terms of OS, the EFS benefit was primarily seen in the FLT3-TKD patients, indicating that the benefit in FLT3-TKD may overestimate the benefit in FLT3-ITD patients. Whether FLT3-ITD patients also benefit from midostuarin is not clearly demonstrated in RATIFY.

Additionally, the authors of RATIFY argue that the benefit of midostaurin persisted when accounting for patients undergoing allo-SCT. While there was a trend toward increased 4-year overall survival in patients who received midostaurin and underwent transplant in 1^st^ CR (CR1) vs. those who received placebo and underwent transplant in CR1, this was not a statistically significant (63.7 vs. 55.7%, p=0.08). In fact, the benefit of midostaurin in transplanted patients was more influenced by timing of transplant. Those patients who received midostaurin and underwent transplant outside of CR1 had a median OS equal to those who received placebo and underwent transplant outside of CR1 (14.8 months vs. 14.4 months). More patients in the midostaurin group underwent transplant in CR1 (28%) than in the placebo group (23%). Thus, the observed benefit of midostaurin may be explained by the fact patients in the midostaurin group underwent transplant earlier.

#### Emerging Clinical Data for the Use of Specific FLT3 Inhibitors in the Upfront Setting

Trials evaluating selective FLT3 inhibitors crenolanib, quizartinib, and gilteritinib in combination with traditional cytotoxic chemotherapy in the upfront setting have shown promising results. In June, 2019, updated results from a phase I/II study evaluating gilteritinib combined with 7 + 3 and consolidation treatment were presented at the annual meeting of the European Hematology Association (EHA) (16). In this study, successive cohorts of patients unselected for FLT3 mutation status received dose-escalating gilteritinib (40, 80, 120, or 200 mg/day) in one of two schedules, in combination with 7 + 3 (Schedule 1: Days 4–17, Schedule 2: Days 8–21). The maximum tolerated dose (MTD) of gilteritinib was determined to be 120 mg which was evaluated in a dose expansion cohort. As of October 2018, 68 patients had enrolled [median age, 59.5 years (range, 23–77)]. Thirty-six patients (54.5%) were *FLT3*^mut+^ (*FLT3*-ITD, n=25; *FLT3*-TKD D835, n=7; *FLT*3-ITD and -TKD D835, n=1; other *FLT3* mutation, n=3). Toxicity was as expected for patients receiving induction therapy. Remarkably, the composite CR rate of *FLT3*^mut+^ patients receiving gilteritinib on Schedule 1 (n=22) was 100% and on Schedule 2 (n=11) was 81.8% with a median DFS of 297 days.

High CR rates have also been observed with 7 + 3 combined with crenolanib (72%) (22) and quizartinib (84%) (23). While these results are early and numbers are small, the high CR rates achieved across the board with selective FLT3 inhibitors certainly surpass the 59% CR rate observed in RATIFY, suggesting that the benefit of selective FLT3 inhibitors is not just in depth of response, as with midostaurin, but that more patients may respond overall.

#### The Argument Against Using a More Specific FLT3 Inhibitor at Relapse for Oligoclonal Disease

During analysis of the ADMIRAL trial, samples from patients who relapsed on gilteritinib were evaluated with next generation sequencing (NGS) for new mutations acquired at relapse (24). Of the 40 patients analyzed, mutations downstream of FLT3 in the Ras/MAPK pathway were the most commonly acquired mutations in 18 (45%) patients. This argues against the theoretical case for “saving” selective FLT3 inhibitors for the time of relapse, as mutations downstream of FLT3 may confer resistance to any FLT3 inhibitors.

The next most common mutations occurring in 6 patients (15%) were mutations in FLT3 itself with the majority (5/6) being FLT3 F691L, or the so called “gatekeeper mutations.” In an *in vitro* analysis by Albers, et al., a patient harboring the FLT3 F691L mutation, while resistant to the selective FLT3 inhibitor quizartinib, retained sensitivity to multikinase inhibitors midostaurin, sunitinib and sorafenib (25). Additionally, Williams, et al. have shown that samples from patients with FLT F691L mutation retain sensitivity to multikinase inhibitors, specifically lestaurtinib and midostaurin (26). These data suggest that, potentially, FLT3 F691L-mutated disease could be successfully treated with midostaurin in the relapse setting.

## Conclusion

Midostaurin remains the only FLT3 inhibitor FDA approved for the upfront treatment of FLT3 mutated AML. The data for use of newer, more selective inhibitors in treatment-naïve patients is rapidly evolving, and soon we may be forced to choose between several FLT3 inhibitors without randomized data directly comparing their clinical efficacy. Here, we have discussed the merits of these two approaches and the data to support them. At our institution, we are currently using midostaurin in addition to 7 + 3 for upfront management of FLT3-ITD AML. We look forward to the release of the final phase II date with gilteritinib in this setting. Further investigation is needed to determine the optimal sequencing of therapies for these complex patients.

## Author Contributions

GK and KLA wrote and edited the manuscript. NDP reviewed and edited the manuscript. All authors contributed to the article and approved the submitted version.

## Conflict of Interest

The authors confirm that the research was conducted in the absence of any commercial or financial relationships that could be construed as a potential conflict of interest.

The handling editor is currently organizing a Research Topic with one of the authors NDP.
